# How do early-life factors explain social inequalities in adolescent mental health? Findings from the UK Millennium Cohort Study

**DOI:** 10.1136/jech-2019-212367

**Published:** 2019-09-06

**Authors:** Viviane S Straatmann, Eric Lai, Theis Lange, Melisa Claire Campbell, Sophie Wickham, Anne-Marie Nybo Andersen, Katrine Strandberg-Larsen, David Taylor-Robinson

**Affiliations:** 1 Public Health and Policy, Institute of Psychology Health and Society, University of Liverpool, Liverpool, UK; 2 Center for Statistical Science, Peking University, Beijing, China; 3 Department of Biostatistic, University of Copenhagen, Copenhagen, Denmark; 4 Department of Public Health, Section of Social Medicine, University of Copenhagen, Copenhagen, Denmark

**Keywords:** SOCIAL INEQUALITIES, MENTAL HEALTH, Lifecourse / Childhood Circumstances, EPIDEMIOLOGY

## Abstract

**Background:**

Reducing inequalities in adolescent mental health is a public health priority, yet the pathways that link social conditions to mental health outcomes in the early years are unclear. We aimed to evaluate the extent to which early years risk factors explain social inequalities in adolescent mental health in the UK.

**Methods:**

We analysed data from 6509 children captured in the UK Millennium Cohort Study. Mental health was assessed through the socioemotional behavioural problems at age 14 (Strengths and Difficulties Questionnaire). The main exposure was maternal education at birth, used as a measure of childhood socioeconomic conditions (SECs), and used to calculate the relative index of inequality. Using causal mediation analysis, we assessed how perinatal, individual child, family, peer relation and neighbourhood-level factors measured up to age 3-mediated the total effect (TE) of SECs on adolescent socioemotional behavioural problems, estimating the proportion mediated and natural indirect effect (NIE) via each block of mediators, and all mediators together.

**Results:**

Children of mothers with no qualification were almost four times as likely to have socioemotional behavioural problems compared with degree plus level (relative risk (RR) 3.82, 95% CI 2.48 to 5.88). Overall, 63.9% (95% CI 50.2% to 77.6%) (NIE RR 1.97, 95% CI 1.63 to 2.37) of the TE (RR 4.40, 95% CI 3.18 to 6.07) of social inequalities on risk of adolescent socioemotional behavioural problems was mediated by early-life factors.

**Conclusions:**

About two-thirds of the social inequality in adolescent mental health was explained by early risk factors measured by age 3, highlighting the importance of public health interventions in this period.

## Introduction

Adolescent mental health is poor in the UK, and there are concerning indications that the situation may be deteriorating, with UK universities reporting a dramatic rise in students reporting mental health conditions over recent years.^1^ There are stark inequalities in mental health, with the most disadvantaged children experiencing worse mental health and subsequent consequences over the course of their lives.^2 3^ A total of 10%–20% of children and adolescents suffer from mental disorders worldwide, and half of all mental illnesses initially manifest by 14 years of age.^4^ In the UK, according to the most recent population-level data,^2^ one in eight children aged 10–15 reported socioemotional behavioural problems in 2011–2012.

Inequalities in mental health outcomes are evident very early in childhood.^5^ Furthermore, we have previously shown that socioemotional behavioural problems at age 11 years can be predicted using data routinely collected in the first 3 years of life, and that socioeconomic factors were the most important predictors.^6^ A recent systematic review of the association between socioeconomic conditions (SECs) and child mental health outcomes found that one in five children experience poor mental health, and those living in disadvantaged childhood SECs were approximately two to three times more likely to develop mental health problems than their peers from more socioeconomically advantaged families. The authors also found that 52 (out of 55) studies reported a graded inverse relationship between SEC and child mental health outcomes, whereby lower socioeconomic status is associated with greater adverse mental health outcomes.^7^ It is essential to understand the early-life drivers of inequalities in adolescent mental health and to unpick causal pathways in order to inform prevention efforts.^1^

While the association of adverse SECs with worse child mental health is well established, we lack understanding of the complex pathways linking social conditions to mental health outcomes.^1 8^ There are a number of plausible mechanisms: children growing up in disadvantaged SECs may be exposed to more traumatic events and stressors (eg, witnessing violence and frequent moves), which in turn may increase their risk of mental and behavioural problems^7–9^; the strain of financial stress may also lead to family conflict and potential disruption (eg, divorce or separation of parents) or may influence parenting behaviours, including increased use of harsh discipline methods, lack of affection and support, or inadequate supervision.^8–10^ In addition, children growing up in disadvantaged SECs may be more likely to reside in more disadvantaged neighbourhoods with higher levels of crime, exposing them to suboptimal physical and social–environmental conditions that may adversely influence their mental health.^9^

A number of studies suggest that early-life risk factors, such as perinatal risks and parental mental health, are predictive of mental health problems in later childhood.^6 11^ However, few studies have explored the extent to which these factors mediate inequalities in mental health in later life. Mäntymaa and colleagues categorise risk factors for child psychopathology as (1) risks in the infant or child, (2) risks affecting the parents, and (3) risks in the family and social context.^10^ However, the interplay of these mechanisms in the early years, as well as their differential impact by SECs, is poorly understood. The rationale for this study was therefore to explore the social gradient in poor adolescent mental health, and the extent to which it is explained by preschool risk factors, using a contemporary UK cohort. This is important from a public mental health policy perspective, since if a large proportion of social inequalities are explained by early years factors, this may guide the timing of interventions and policies to reduce inequalities in mental health across the life course. We hypothesised that children growing up in more disadvantaged circumstances are at increased risk of adolescent mental health problems due to increased exposure to risk factors in early life, and that modifiable causal pathways to health inequalities in adolescent mental health can be identified in order to guide policy and practice.

## Methods

### Study design and population

The Millennium Cohort Study (MCS) is a large nationally representative cohort sample study of 18 818 children born in the UK between 2000–2002. To date it includes six sweeps: 9 months, 3, 5, 7, 11 and 14 years old, and we used data from 9 months, 3 and 14 years old. We included all singleton children with complete data provided by the main respondent (almost always the mother). The study oversampled children living in disadvantaged areas and in ethnic minority groups by means of a stratified cluster sampling design.^12^

### Measures

#### Mental health outcome

Our outcome was adolescent socioemotional behavioural problems assessed using maternal reported Strengths and Difficulties Questionnaire (SDQ) when the cohort participants were aged 14 years. The SDQ is a widely used and validated screening tool to measure emotional and behavioural problems (ie, overall mental health) in a number of settings.^13^ The SDQ is a 25-item measure that asks parents to rate their child’s behaviour over the previous 6 months using five subscales, each with five items: peer problems, conduct disorders, hyperactivity, emotional problems and prosocial behaviour. We used the total difficulties score (which excludes the prosocial behaviour items) using a validated cut-off widely used in previous studies.^14 15^ A score of 0–16 indicates ‘normal to borderline behaviour’, and 17–40 indicates ‘socioemotional behavioural problems’.^16^

### Measurement of socioeconomic circumstances (SECs)

Our primary exposure of interest was the highest qualification attained by the mother around the time of MCS child's birth, used as a measure of childhood SECs at the birth of the cohort child: (1) ‘degree plus=higher degree or first degree qualifications’; (2) ‘diploma=in higher education’; (3) ‘A levels’ (exams taken around 18 years); (4) ‘General Certificate of Secondary Education (GCSE, exams taken around age 16 years) grades A–C’; (5) ‘GCSE grades D–G’; and (6) ‘None of these qualifications’. It was coded as a categorical variable for the first step of our analyses. Level of maternal educational qualifications is a commonly used measure of childhood SECs in social epidemiological studies^17 18^ and represents a more stable measure of SECs as compared with income, which could be fluctuated at times. It also encompasses a range of non-economical social attributes, for example, general and health-related knowledge, literacy and problem-solving skills; prestige; and influence over others and one's own life.^19^ The online supplementary material provides more information about the education system in the UK (online supplementary material S1). Robustness test using income as the main exposure was also performed (details as follows).

10.1136/jech-2019-212367.supp1 Supplementary data

In the second step, we calculated the relative index of inequality (RII), which compares the risk of mental health problems between children of lowest and highest socioeconomic status, taking into account the educational distribution, by ranking the six maternal educational groups from the lowest to the highest and allocating a score (ranging from 0 to 1) that equals the midpoint of the category’s range in the cumulative distribution. The RII is a regression-based index that summarises the relative inequality across the distribution of SECs, taking into account the size of the population and the relative disadvantage experienced by different groups. For instance, if 24% of the mothers had no formal education, they would be allocated a score of 0.12, and if the next group of mothers constituted 42%, they would be allocated a score of 0.45 (0.24+0.42/2). Using this score as a continuous exposure variable in the regression model, its estimated coefficient expresses the RII, with a similar interpretation to a relative risk (RR).^20^

### Potential mediating risk factors

**Figure 1:**
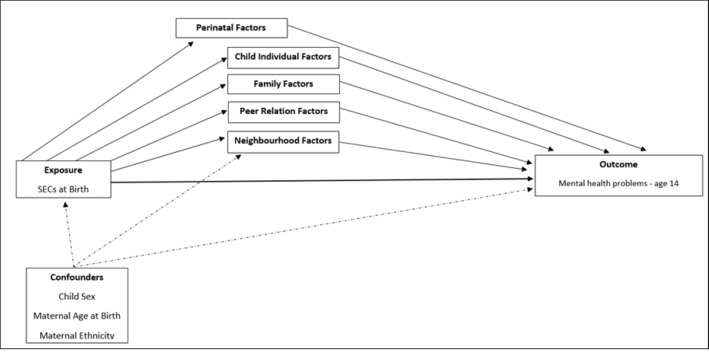
Logical model of block of perinatal and early-life (age 3 years) mediators of SECs and maternal report of mental health problems at age 14 years. SEC, socioeconomic condition.

We identified in literature reviews five potential blocks of early childhood/preschool risk factors for social gradient in adolescent mental health,^7–11^ and mapped these onto data available on the first and second sweeps of the MCS. The potentially mediating risk factor blocks were ordered from proximal to distal influences in the child^4^ (figure 1) and were reported by the main responders. The full details of the coding of these mediators are provided in the box 1 and in the online supplementary material S2.

Box 1 **Description of blocks of potential mediating risk factors**

- Perinatal factors
  - Maternal smoking in pregnancy (first sweep, at birth): the main responder was questioned about ‘Gave up during pregnancy or did not smoke immediately prior to pregnancy’—‘yes’ versus ‘no’.
  - Alcohol consumption in pregnancy (first, sweep at birth): the main responder was questioned about ‘Have you drank any alcohol during pregnancy?’—‘yes’ versus ‘no’.
  - Gestational age (first sweep, at birth): the main responder was asked about the gestational age at birth (‘preterm <37 weeks’ vs ‘term ≥38 weeks’).
  - Breast feeding (first sweep, at birth): the main responder was asked about the duration of the breast feeding (‘less than 4 months’ vs ‘at least 4 months’).
  - Birth weight (first sweep, at birth): the main responder was asked about the birth weight of the child (low birth weight <2.5 kg vs normal ≥2.5 kg).
- Child individual factors
  - School readiness (second sweep, age 3): Bracken School Readiness Assessment* was assessed and coded as ‘average/advanced’ versus ‘delayed/very delayed’.
  - Long-term disabilities or illness (second sweep, age 3): The main responder was questioned whether the child had any disabilities or long-term illness (‘no’ vs ‘yes’).
  - Cognition (second sweep, age 3): British Ability Scale Second Edition (BAS II) Naming and Vocabulary† was assesed; we defined children as having language disability whether they scored –1.25 SDs below the normed mean score, for the sample.
- Family factors
  - Maternal mental health (second sweep, age 3): Kessler 6 scale was used to assess maternal mental health in the last month, asking the responders how often they felt depressed, hopeless, restless or fidgety, worthless, or that everything was an effort. Respondents answered on a 5-point scale from 1 (all the time) to 5 (none of the time). We reversed and rescaled all items from 0 to 4 for analysis purposes, so that high scores indicated high levels of psychological distress (‘mental illness: 6–24 scores= yes/no’).‡
  - Parenting style (second sweep, age 3): it was assessed asking the main responder about her/his style of parenting and coded as ‘firm discipline plus fun’ versus ‘education negligence or excess of rules’.
  - Child–parents conflict relationship (second sweep, age 3): the main responder answered a 7-item scale§ about her/his relationship with the child in terms of conflicts (‘lowest conflict, score 7–15’ vs ‘highest conflict, score 16–40’).
  - Lone parenthood (first sweep, at birth): the main responder was asked whether her/his has been in a lone parenthood (‘yes’ vs ‘no’).
- Peer relation factors
  - Time spent with friends (second sweep, age 3): the main responder was asked about the amount of time that the child spends with friends (‘spend any time per week with friends’ vs ‘not at all’).
  - Being bullied (second sweep, age 3): the main responder was questioned whether the child has been bullied (‘not being bullied’ vs ‘some true or certainly true’).
  - Bullying other peers (second sweep, age 3): the main responder was questioned whether the child fights or bullies’ other peers (‘not fights or bullies’ vs ‘some true or certainly true’).
- Neighbourhood factors
  - Neighbourhood conditions (first sweep, at birth): the main responder was asked about the general conditions of her/his neighbourhood (‘not very common or not at all common neighbourhood problems’ vs ‘fairly or very common neighbourhood problems’).
  - Neighbourhood safety (second sweep, age 3): the main responder was asked about neighbourhood safety in the living area (‘very safe or fairly safe’ vs ‘not safe’).

*Bracken B. Bracken Basic Concept Scale–Revised. San Antonio, TX: The Psychological Corporation 1998.†Elliott C. The British Ability Scales II. Windsor, Berkshire, 1996, UK: NFER-NELSON Publishing Company.‡Kessler RC, Andrews G, Colpe L, et al. Short screening scales to monitor population prevalence and trends in non-specific psychological distress. Psychol Med. 2002; 32: 959–76.§Pianta RC. Child‐Parent Relationship Scale. Charlottesville: University of Virginia, 1995.

### Analysis and statistical modelling

We estimated the prevalence of mental health problems at age 14 by maternal education and tested univariate associations between our mediators of interest and child mental health. Then, the analysis progressed in two stages. First, we ran sequentially adjusted Poisson regression models to assess how the RR for the association between childhood SECs and child mental health changed on adjustment for the blocks of potentially mediating factors, added individually, and then all together. We adjusted for potential confounders, as guided by a directed acyclic graph (figure 1). Maternal ethnicity was considered a potential baseline confounder, as it may influence both SEC and adolescent health. We evaluated the change in RRs comparing mothers with the highest qualifications with those with the lowest calculated as 100×(RR−adjustedRR)/(RR−1). Sampling and response weights were used to account for the sampling design and attrition.

Second, we undertook a counterfactual mediation analysis to formally assess the amount of social inequality in mental health at age 14, explained by each mediating block, using the RII as the exposure. We estimated the RRs and 95% CI for the natural direct effect (NDE), natural indirect effect (NIE) and total effect (TE) (formulas and definitions in the online supplementary material S3) for each block of mediators individually and all blocks together using the medflex package in R software. This package fits natural effect models, a novel class of counterfactual models to directly parameterise the path-specific effects of interest, in the presence of multiple mediators, taking into account interactions between the variables included in the mediating blocks.^21^ We calculated the proportion mediated and the 95% CI for each block of mediators by applying the formula: (RR^NDE^×(RR^NIE^−1))/(RR^NDE^×RR^NIE^−1).^22^ All analyses were conducted in Stata/SE V.15 and R V.3.4.4.

### Robustness tests

We used multiple imputation by chained equation^23^ in order to check whether there are differences in descriptive and associative results of complete cases and imputed samples. We did additional descriptive analysis comparing baseline cases and complete cases for child sex, maternal ethnicity and maternal education.

We repeated our first step of regression analysis using RII as the exposure variable for the purpose of comparison with the counterfactual mediation analysis. Although we have included factors from proximal to distal relation to the child observing the separate effect of each block and, finally, the combined effect of all, we repeated step 1 of our analyses, taking into account a probabilistic chain effect (ie, dosage, context and timing) of the perinatal block in the subsequent ones.^4^ Although we aimed to assess overall mental health difficulties, we developed an additional analysis using depressed mood symptoms reported by the adolescents at age 14, assessed by the short version of the Mood and Feelings Questionnaire. We also performed analyses including previous diagnosis of maternal mental health problems. To explore exposure–mediator interaction, we repeated the analysis allowing for all two-way interactions between maternal education and the mediators in the model and used Akaike Information Criterion (AIC) to compare the model fit. We tested for potential bias in estimates of direct and indirect effects due to confounding of the mediator–outcome relationship.^22 24 25^ The counterfactual mediation analysis was also repeated using equivalised family income as an alternative measure of childhood SECs.

## Results

A total 10 264 children who participated in the first (9 months) and the latest (14 years) sweeps had socioemotional behavioural data at age 14. Data on cohort members’ mental health (main outcome) and maternal education at birth (main exposure) were available for 9962 participants. Around two-thirds (n=6509) had full data on all exposure, outcome, mediators and confounders of interest, that is, the complete case population.

**Figure 2:**
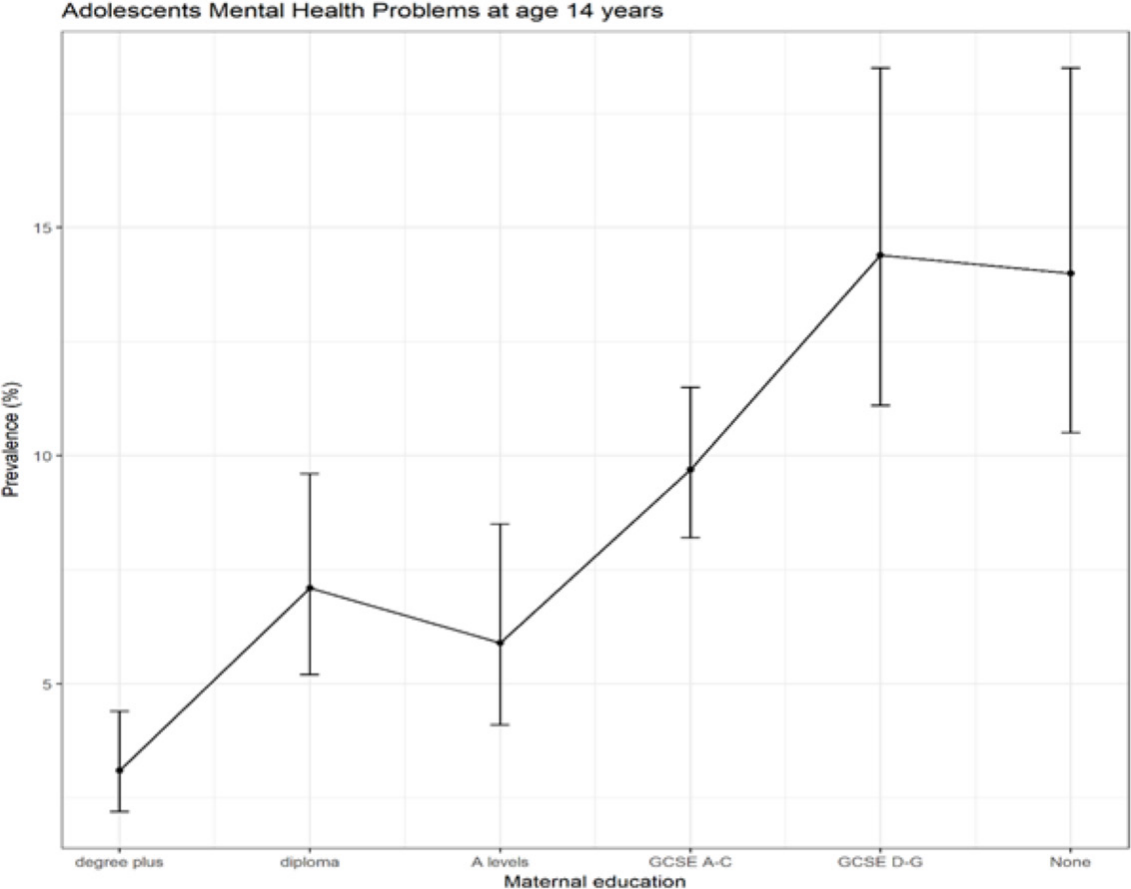
Prevalence (%) and CIs (95% CI) of adolescents’ mental health problems in the UK at age 14 by maternal education at birth (N=6509). GCSE, General Certificate of Secondary Education.

Nine per cent (95% CI 7.9% to 10.0%, mean score 8.1 (±5.9)) of children had mental health problems by 14 years. There was a clear social gradient in socioemotional behavioural problems, whereby the proportion of children reporting problems increased as childhood SEC level decreased, as measured by maternal educational qualification level (figure 2). All characteristics of the study population, except child sex and long-term disability or illness at age 3, were associated with childhood SECs (table 1).

**Table 1:** Characteristics of the complete case study population by level of maternal education at birth of child (N=6509)

	Degree plus	Diploma	A levels	GCSE A–C	GCSE D–G	None	Total	P value
	%	%	%	%	%	%	%	
Adolescents socioemotional behavioural problems at age 14	3.1	7.0	5.9	9.7	14.3	14.4	8.9	<0.001
Child’s sex								0.701
Male	50.2	51.2	46.2	50.2	48.3	47.9	49.6	
Maternal age at MCS birth								<0.001
14–24 years	26.5	41.5	47.5	52.6	71.4	61.3	47	
Maternal ethnicity								<0.001
Mixed	0.8	0.6	0	0.5	0.5	0.6	0.5	
Indian	2.0	1.4	1.2	0.9	1.4	2.0	1.4	
Pakistani	0.9	0.9	1.6	1.5	1.4	4.3	1.6	
Bangladeshi	0.1	0.4	0.3	0.2	0.4	1.0	0.3	
Black	2.4	2.3	0.5	0.6	1.5	0.8	1.2	
Other	1.4	0.7	1.0	0.3	0.1	1.0	0.7	
Smoking in pregnancy								<0.001
Yes	3.4	9.9	11.1	20.9	33.7	46.3	^17^.7	
Alcohol consumption in pregnancy								<0.001
Any unit per week	65.3	51.6	46	40.3	30.1	26.3	46.1	
Gestational age at birth								0.007
Preterm	4.6	5.8	3.4	5.9	5.8	6.7	5.4	
Child’s birth weight								<0.001
Low weight	3.6	5.4	4.7	5.5	6.4	8.9	5.3	
Breast feeding at least 4 months								<0.001
No	35.7	59.7	58.1	73.1	82.1	84.2	62.8	
Cognitive disability at age 3								<0.001
Yes	1.5	2.3	2.3	4	5.7	11.1	3.8	
School readiness at age 3								<0.001
Very delayed or delayed	3.4	3.9	4.9	8.6	14.6	20.3	7.9	
Child’s long-term disabilities or illness at age 3								0.063
Yes	14.3	14.8	11.3	15.4	16.3	19.4	15	
Maternal mental health problems								<0.001
Yes	10.9	12.7	13.5	18.4	18.4	29.4	16.3	
Parenting style								<0.001
Negligent or harsh parenting style	40.1	43.7	45.4	52.1	58	62.6	49	
Child–parents conflict relationship								0.001
High conflicts	52.5	52.5	52.2	52.3	54.5	63.5	53.5	
Lone parenthood								<0.001
Yes	1.7	4.4	8	12.5	16.3	23.8	9.8	
Child’s time spent with friends								0.027
Not at all	0.6	0	1.5	0.6	0.6	1.3	0.7	
Being bullied								<0.001
Some true or certainly true	4.3	3.9	5.7	6.5	10.5	13.4	6.5	
Fights or bullies’ other peers								<0.001
Some true or certainly true	9.3	10.7	11.6	14.4	21.6	30.4	15.9	
Neighbourhood conditions								<0.001
Fairly common or very common neighbourhood problems	50	55.9	56.3	60.2	70.8	72.6	58.9	
Neighbourhood safety								<0.001
Fairly safe	5.1	6.7	9.5	14.5	18.1	23.6	13	

Reference categories were omitted. Full table annexed in online supplementary material S6.

*online supplementary material S6

MCS, Millennium Cohort Study.

In table 2, the prevalence of socioemotional behavioural problems at age 14 and univariate associations are presented (full description in online supplementary material S4).

**Table 2:** Prevalence of socioemotional behavioural problems at age 14 and univariate RRs (N=6509)

	Total %	Adolescent socioemotional behavioural problems %	RR (95% CI)
Maternal education			
Degree plus	6.4	3.1	1.00
Diploma	7.6	7.1	2.26 (1.44 to 3.54)
A levels	6.7	5.9	1.88 (1.13 to 3.15)
GCSE A–C	41.2	9.7	3.09 (2.10 to 4.55)
GCSE D–G	17.5	14.4	4.60 (2.98 to 7.09)
None	20.6	14	4.48 (2.91 to 6.88)
Child’s sex			
Male	53.9	9.7	1.18 (0.95 to 1.48)
Female	46.1	8.2	1.00
Maternal age at MCS birth (years)			
14–24	67.8	11.5	1.93 (1.55 to 2.41)
25+	32.2	6.1	1.00
Maternal ethnicity			
White	93.5	8.3	1.00
Mixed	0.5	26.1	1.17 (0.55 to 2.47)
Indian	1.8	2.3	0.41 (0.24 to 0.68)
Pakistani	2.0	12.4	1.19 (0.91 to 1.53)
Bangladeshi	0.5	0	0.82 (0.60 to 1.13)
Black	0.9	10.4	1.03 (0.68 to 1.56)
Other	0.7	3.3	0.62 (0.22 to 1.78)
Smoking in pregnancy			
No	62.2	7.2	1.00
Yes	37.8	15.1	2.06 (1.66 to 2.54)
Alcohol consumption in pregnancy			
None	64.6	10.4	1.00
Any unit(s) per week	35.4	7.1	0.69 (0.56 to 0.86)
Gestational age at birth			
Preterm	6.2	9.8	1.11 (0.75 to 1.65)
Regular term	93.8	8.9	1.00
Child’s birth weight			
Low weight	92.5	11.5	1.34 (0.91 to 1.96)
Normal+	7.5	8.8	1.00
Breast feeding at least 4 months			
Yes	19	5.3	1.00
No	81	10.7	2.08 (1.59 to 2.73)
Cognitive disability at age 3			
No	90.3	8.5	1.00
Yes	9.7	19.7	2.33 (1.65 to 3.30)
School readiness at age 3			
Average, advanced or very advanced	83.7	8.3	1.00
Very delayed or delayed	16.3	14.8	1.72 (1.26 to 2.34)
Child’s long-term disabilities or illness at age 3			
No	76.5	8.1	1.00
Yes	23.5	13.8	1.73 (1.34 to 2.23)
Maternal mental health problems			
No	67.5	7.3	1.00
Yes	32.5	16.5	2.25 (1.81 to 2.79)
Parenting style			
Firm discipline plus fun	44.9	8.1	1.00
Education negligence or excess of rules	55.1	9.8	1.19 (0.98 to 1.44)
Child–parents conflict relationship			
Low conflicts	23.2	4.7	1.00
High conflicts	76.8	12.2	2.61 (2.08 to 3.27)
Lone parenthood			
No	76.6	7.9	1.00
Yes	23.4	15.4	1.95 (1.49 to 2.54)
Child’s time spent with friends			
Any time per week with friends	98.7	8.9	1.00
Not at all	1.3	16.4	1.86 (0.86 to 4.01)
Being bullied			
Not being bullied	86.3	8.4	1.00
Some true or certainly true	13.7	16.2	1.89 (1.28 to 2.80)
Fights or bullies other peers			
Not fights or bullies	65.1	6.9	1.00
Some true or certainly true	34.9	19.5	2.76 (2.17 to 3.50)
Neighbourhood conditions			
Not at all or nor very common neighbourhood problems	26.7	6.0	1.00
Fairly or very common neighbourhood problems	73.3	10.9	1.84 (1.45 to 2.34)
Neighbourhood safety			
Very safe	80.9	8.3	1.00
Fairly safe	19.1	12.9	1.60 (1.19 to 2.15)

GCSE, General Certificate of Secondary Education; RR, relative risk.

Table 3 shows the distribution of the sample in terms of maternal education, and the extent to which the elevated RR of socioemotional behavioural problems in 14-year-old adolescents with mothers with no qualifications was attenuated when adjusting separately for each block of mediators. There was a 40.8% reduction to aRR 2.67 (95% CI 1.68% to 4.23%) adjusting for perinatal factors (model 1); a 12.7% reduction (aRR 3.46, 95% CI 2.22% to 5.39%) adjusting for child factors (model 2); a 25.8% (aRR 3.09, 95% CI 1.96% to 4.89%) and 26.9% (aRR 3.06, 95% CI 2.00% to 4.58%) reduction for family (model 3) and peer relation factors (model 4) respectively; and a 13.8% reduction adjusting for neighbourhood factors (model 5, aRR 3.43, 95% CI 2.16% to 4.94%). In model 6, adjusted for all blocks, the RR was attenuated by 64.8% (aRR 1.99, 95% CI 1.22% to 3.26%).

**Table 3:** Regression models for socioemotional behavioural problems at age 14 and covariate estimates using complete case analysis (N=6509)

			RR (95% CI)*				
	Baseline†	Model 1	Model 2	Model 3	Model 4	Model 5	Model 6
Maternal education							
Diploma	2.08 (1.32 to 3.27)	1.88 (1.19 to 2.98)	2.06 (1.31 to 3.22)	2.09 (1.33 to 3.29)	2.06 (1.26 to 3.05)	2.04 (1.22 to 2.97)	1.89 (1.20 to 3.00)
A levels	1.71 (1.03 to 2.85)	1.55 (0.92 to 2.59)	1.71 (1.03 to 2.84)	1.68 (1.01 to 2.78)	1.68 (1.01 to 2.80)	1.66 (1.00 to 2.75)	1.55 (0.93 to 2.60)
GCSE A–C	2.70 (1.83 to 3.98)	2.21 (1.46 to 3.33)	2.60 (1.76 to 3.83)	2.50 (1.68 to 3.72)	2.57 (1.64 to 3.33)	2.52 (1.60 to 3.27)	2.02 (1.32 to 3.11)
GCSE D–G	3.73 (2.41 to 5.77)	2.79 (1.74 to 4.49)	3.46 (2.21 to 5.40)	3.39 (2.17 to 5.28)	3.28 (2.15 to 4.85)	3.37 (2.20 to 4.98)	2.36 (1.44 to 3.87)
None	3.82 (2.48 to 5.88)	2.67 (1.68 to 4.23)	3.46 (2.22 to 5.39)	3.09 (1.96 to 4.89)	3.06 (2.00 to 4.58)	3.43 (2.16 to 4.94)	1.99 (1.22 to 3.26)
Perinatal factors							
Smoking in pregnancy		1.47 (1.12 to 1.92)	–	–	–	–	1.23 (0.94 to 1.61)
Alcohol consumption in pregnancy		0.92 (0.74 to 1.13)	–	–	–	–	0.90 (0.74 to 1.10)
Gestational age at birth		0.95 (0.65 to 1.38)	–	–	–	–	0.87 (0.58 to 1.29)
Child’s birth weight		1.16 (0.77 to 1.76)	–	–	–	–	1.17 (0.78 to 1.75)
Breast feeding at least 4 months		1.35 (1.01 to 1.80)	–	–	–	–	1.21 (0.91 to 1.61)
Child individual factors							
Cognitive disability		–	1.64 (1.11 to 2.41)	–	–	–	1.45 (0.99 to 2.12)
School readiness		–	1.15 (0.82 to 1.62)	–	–	–	1.01 (0.73 to 1.40)
Child’s long-term disabilities or illness		–	1.59 (1.24 to 2.04)	–	–	–	1.47 (1.15 to 1.87)
Family factors							
Maternal mental health problems		–	–	1.61 (1.33 to 3.29)	–	–	1.52 (1.19 to 1.93)
Parenting style		–	–	1.00 (0.81 to 1.23)	–	–	1.02 (0.83 to 1.25)
Lone parenthood		–	–	1.27 (0.95 to 1.71)	–	–	1.15 (0.85 to 1.55)
Child–parents conflict relationship		–	–	2.20 (1.70 to 2.84)	–	–	1.85 (1.43 to 2.39)
Peer relation factors							
Child’s time spent with friends		–	–	–	1.97 (0.95 to 3.90)	–	1.70 (0.91 to 3.20)
Being bullied		–	–	–	1.35 (0.94 to 1.96)	–	1.15 (0.79 to 1.67)
Fights or bullies other peers		–	–	–	2.22 (1.75 to 2.83)	–	1.76 (1.39 to 2.23)
Neighbourhood factors							
Neighbourhood conditions		–	–	–	–	1.51 (1.18 to 1.94)	1.35 (1.07 to 1.72)
Neighbourhood safety		–	–	–	–	1.20 (0.88 to 1.65)	1.05 (0.78 to 1.41)
Proportion attenuated (%)‡		40.8	12.7	25.8	26.9	13.8	64.8

*All models were adjusted for baseline confounders (maternal age birth, child sex and maternal ethnicity)—omitted table results.

†Adjusted only for baseline confounders—omitted table results.

‡Proportion of RR attenuated by comparison of baseline model with models 1–6.

GCSE, General Certificate of Secondary Education; RR, relative risk.

**Figure 3:**
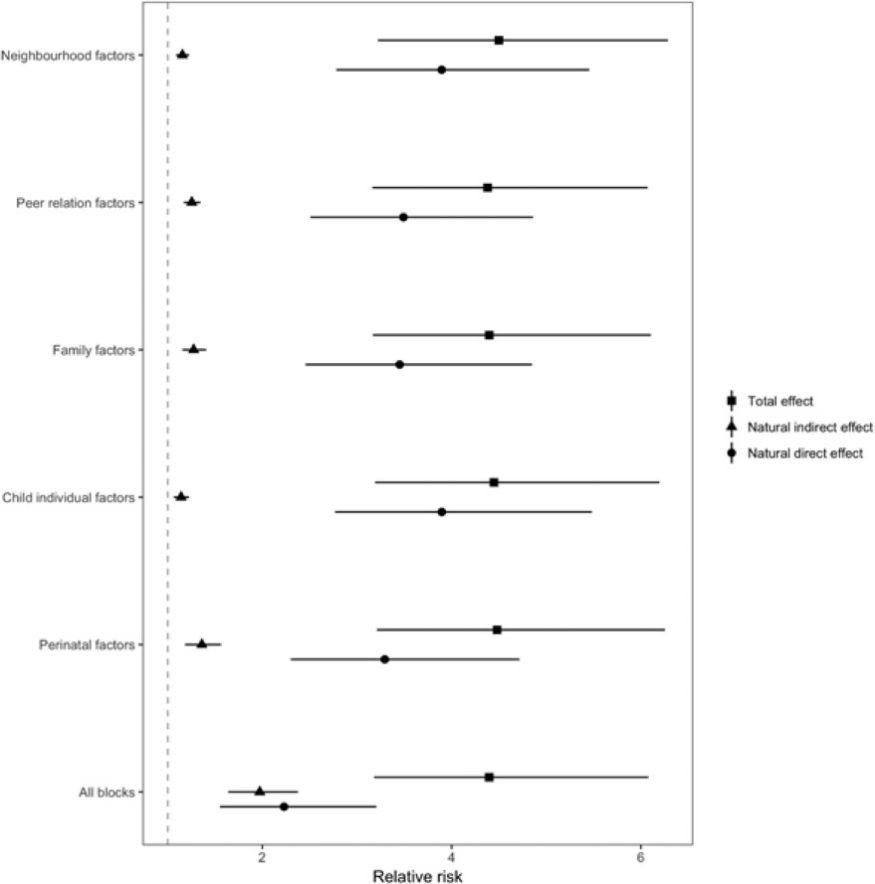
Mediation analysis with a conterfactual approach by block of risk factors (perinatal, child individual, family, peer relations and neighbourhood) in the association between socioeconomic condition and adolescents’ mental health at age 14 (relative risk and CIs (95% CI)) (N=6509).

The counterfactual mediation analysis results are shown in figure 3 and detailed in table 4 (log-RR results are presented in online supplementary material S5). The TE of a hypothetical intervention changing all from high SEC to low SEC (hypothetical at the bottom of the educational hierarchy) on children’s mental health had an RR of 4.40 (95 %CI 3.18 to 6.07). The natural direct effect (RR 2.23, 95% CI 1.55 to 3.20) is the increase in socioemotional behavioural problems risk, comparing low to high SEC that we would observe if the mediators remained as in the top end of the SEC hierarchy; and the NIE is the increased risk of socioemotional behavioural problems we would see if the SECs were fixed at top of the SEC hierarchy, but the mediators were fixed at those that would naturally occur at low SECs (RR 1.97, 95% CI 1.63 to 2.37). These RRs can also be expressed as proportion mediated, which overall yields 34% (95% CI 19.9% to 47.5%), 16% (95% CI 7.8% to 24.2%), 28% (95% CI 17.2% to 38.3%), 26% (95% CI 18.8% to 34.2%) and 17% (95% CI 10.3% to 24.2%) of the TE of SECs on risk of socioemotional behavioural problems at age 14 years in UK children, through exposure to perinatal, child individual, family, peer relations and neighbourhood factors, respectively. Considering all blocks of early risk factors together, 63.9% (95% CI 50.2% to 77.6%) of the TE of SEC on risk of adolescent socioemotional behavioural problems was mediated. It is noted that interpretation as ‘effect of a hypothetical intervention’, as well as the inclusion of mediators as a block, critically hinges on our use of the counterfactual framework.

**Table 4:** NDE, NIE, TE and proportion mediated for exposure maternal education at birth mediated by blocks of risk factors for adolescents’ socioemotional behavioural problems at age 14 (N=6509)

Blocks of mediators	Effect	RR (95% CI)	Proportion mediated, % (95% CI)
Perinatal	NDE	3.29 (2.29 to 4.71)	34.1 (19.9 to 47.5)
	NIE	1.36 (1.18 to 1.56)	
	TE	4.48 (3.21 to 6.25)	
Child individual	NDE	3.90 (2.76 to 5.48)	16.1 (7.8 to 24.2)
	NIE	1.14 (1.06 to 1.22)	
	TE	4.45 (3.19 to 6.19)	
Family	NDE	3.45 (2.45 to 4.89)	27.9 (17.2 to 38.3)
	NIE	1.30 (1.15 to 1.40)	
	TE	4.40 (3.16 to 6.10)	
Peer relation	NDE	3.50 (2.50 to 4.86)	26.4 (18.8 to 34.2)
	NIE	1.25 (1.17 to 1.34)	
	TE	4.38 (3.16 to 6.07)	
Neighbourhood	NDE	3.89 (2.78 to 5.45)	17.3 (10.3 to 24.2)
	NIE	1.15 (1.08 to 1.22)	
	TE	4.49 (3.22 to 6.28)	
All blocks	NDE	2.23 (1.55 to 3.20)	63.9 (50.2 to 77.6)
	NIE	1.97 (1.63 to 2.37)	
	TE	4.40 (3.18,6.07)	

NDE, natural direct effect; NIE, natural indirect effect; TE, total effect.

### Robustness tests

The results for step one of our analysis were similar in our multiply imputed sample (online online supplementary material S7–S9). The baseline in SEC association was RR 4.57, 95% CI 3.44 to 6.06, after adjustment for confounders), and this was attenuated by 57% to 2.20 (95% CI 1.54% to 3.14%) in the final model. Baseline cases and complete cases sociodemographic characteristic are shown in online supplementary material S10). Results using a probabilistic chain approach are presented in online supplementary material S11. Results using a probabilistic chain approach were presented in online supplementary material S12. There was a higher percentage of adolescents with depressed mood comparing adolescents with mothers with none educational qualifications (21.4%) compared with adolescents with mothers with degree plus level education (13%) (online supplementary material S13). Repeating the first step of our analysis using depressed mood as the outcome showed that adjusting for early-life risk factors attenuated the inequality gap by 66.6% (online supplementary material S14). Repeating the analysis including information on previous diagnosis of maternal mental health problems did not change the results (results not shown).

For the counterfactual mediation analysis, a model that included all exposure–mediator interactions had a worse fit based on AIC (results not shown). Simulated scenarios of unmeasured mediator–outcome confounding did not change substantially our estimates (online supplementary material S15). Repeated counterfactual analysis using RII on the bases of income presented similar results’ pattern of maternal education, supporting the use of the latest as a measure of SEC in childhood (online supplementary material S16).

## Discussion

Using nationally representative data, we showed that around 1 in 10 young people born in the new millennium have mental health problems by age 14 in the UK. There were stark social inequalities whereby the risk of mental health problems was around four times higher for children growing up in adverse SECs compared with highest SECs. Around two-thirds of this increased risk was explained by early years risk factors identified by the age of 3 years, related to perinatal, child, family, peer relations and neighbourhood characteristics.

The inequalities in adolescent mental health identified in our study corroborated a systematic review of 55 studies that demonstrated an inverse relationship between socioeconomic status and mental health problems in children/adolescents. In this study, youths in socioeconomically disadvantaged populations were two to three times more likely to develop mental health problems.^7^ In addition, there is evidence on the cumulative impact of childhood socioeconomic deprivation on mental health in adolescence, reinforcing the hardship of deprivation is even worse when accumulated throughout early childhood and school age.^26^

Previous studies suggest that early-life risk factors, such as perinatal risks and parental mental health, are predictive of mental health problems in later childhood.^6 11^ Furthermore, we have demonstrated that clear inequalities in child mental health are evident by age 3 years.^5^ Our study builds on this evidence base, demonstrating the extent to which early-life risk factors mediate inequalities in later adolescent mental health. We showed that about two-thirds of the inequality in mental health outcomes in adolescence can be explained by early years’ perinatal, child, family and neighbourhood factors.

Understanding the role of early risk factors in explaining inequalities in adolescent mental health is critical to inform effective interventions,^11^ and our study is one of the first to attempt to decompose the contribution of different blocks of risk factors using counterfactual mediation analysis. The prenatal period and the first 2–3 years are crucial periods of maximum brain growth, development and formation of emotional regulatory patterns,^27^ but still have opportunities for intervention to improve the child outcomes.^28^ Even if risk factors (eg, genetic risk or early environmental factors such as severe deprivation) are present, without additional risks, a disorder ultimately might not develop.^11^

Perinatal factors alone were the most influential block in our analysis, mediating 34% of the association of SEC and mental health at age 14 years, with significant associations between maternal smoking in pregnancy and shorter duration of breast feeding, and increased risk of adolescent mental health problems. It is likely that prenatal exposures such as maternal alcohol and tobacco smoking impact adversely on early child neurodevelopment, as suggested in a recent systematic review,^29^ with subsequent impacts on risk of mental health problems later in life.^30^ Other evidence has suggested that shorter duration of breast feeding is an independent predictor of mental health problems through childhood and into adolescence.^31^ Possible mechanisms include effects of breast feeding on neuroendocrine aspects of the stress response,^32^ impacts on attachment^33^ and infant temperament,^34^ and direct effects of maternal milk on neurodevelopment.^35^ In this context, it is difficult to establish precise causal pathways, especially because the ways in which risk factors interplay are very complex, and the many intervening factors make it difficult to isolate effects of a single specific factor.^36^ Family factors alone mediated 27% of the inequality in child mental health, indicating another important target for public health intervention. A systematic review suggested that parental depression, conflict and parenting practices, and factors related to resilience in adolescence are potential mediators of the relationship of socioeconomic status and psychosocial outcomes in adolescence.^8^ A supportive family environment seems to be a key factor in developing children's social and emotional competence skills and in promoting positive parenting and facilitating family communication and problem solving.^11^

Our results pointed that peer relation in early childhood mediated a about 26% of inequalities in mental health at age 14. Although school-based interventions targeting children, parents and education professionals are effective strategies for promoting healthy peer relationships,^11 37^ there is evidence that antibullying interventions should commence in primary school, breaking even earlier the potential chains of developing later mental health problems.^38^ Moreover, neighbourhood aspects play an importance role on preventing stigmatisation and stimulating inclusion of cost-effective prevention programmes for mental disorders in political agendas in a community level^11^; our study showed that it explained about 17% of mental health inequalities at age 14. Lastly, our results showed that child characteristics mediated about 16% of inequalities in mental health; pieces of evidences suggest that language and motor delays, subthreshold hyperactivity, low cognitive performance and decline in IQ may be helpful markers of developmental deviance that can precede severe mental and behavioural disorders throughout life.^11^

### Strengths and limitations

A key strength is that this study used secondary data from a large, contemporary UK cohort, which measures different indicators of SEC. A wide range of information is collected in the MCS, which allowed us to explore a range of preschool risk factors for adolecents mental health problems. This study adds to the literature by being the first study to formally test the mediating role of different risk factors of social inequality of adolescents’ mental health with methodological robustness, using counterfactual methods. The use of a validated measure of adolescent mental health assessed by socioemotional behaviours (SDQ) is also a strength of this study.

A potential limitation of our study is the main responder self-reported nature of the mental health outcome. However, there is a clear social gradient in self-reported mental health problems, partially explained by early years risk factors. In addition, establishing precise causal pathways is challenging. The individual block estimates need to be interpreted with caution, since we decided to look at the separate effect of each block, considering a proximal to distal factors approach rather a probabilist chain. Thus, while the total proportion mediated by all the blocks is relatively robust, the sum of the individual blocks adds up to more than the total, since the blocks are likely to affect one another. It may also be the case that the inequalities are subsequently mediated through measure of mental health at ages prior to 14 years, but this does not impact on our main conclusions about the importance of actions in the early years. While the focus of this paper is on early years risk factors that might explain inequalities in adolescent mental health, subsequent analyses should explore potentially modifiable pathways at subsequent time points, for instance, in the school-age period.

Data about potentially mediating childhood adversities such as sexual abuse and parental criminality were not included in our analysis. We were unable to adjust for genetic risk factors for mental health problems, which may partially explain some intergenerational transfer of risk^39^ and epigenetic influences.^40^ Bullying or being bullied are risk factors for subsequent mental health problems,^38^ and in our analysis, this association was evident even for very early measures of peer relationship problems. Subsequent analyses should explore the impact of peer relationship problems at later ages. Missing data may be a limitation, however, repeating the first stage of our analysis led to similar conclusions when rerunning the analyses in an imputed sample. Sampling and response weights were for the first stage of analysis to account for the sampling design and attrition to age 14; however, these cannot account for item missingness.

### Policy and practice implications

From a public health policy perspective, our results support the need for an early years prevention focus to ensure a safe and healthy pregnancy, a nurturing childhood and support for families in providing such circumstances in which to bring up children. While this is currently advocated in UK mental health strategy,^41^ much of the current action is focused on addressing mental health in schools and improving access to mental health services for children.^1^ While these are clearly of critical importance, our results suggest a platform of early investment is required in order to build the foundations for healthy mental health at the population level. In the UK, it is concerning that funding for early years provision has been disproportionately cut in some of the most disadvantaged areas^42^ and that child poverty, a major socioeconomic determinant of child mental health, is currently increasing.^3 15^

In conclusion, we found that 9% of children had mental health problems by age 14 in a nationally representative UK child cohort. The risk was much greater in disadvantaged children, and about two-thirds of this excess risk was explained by early childhood factors up to age 3 years. Future research should investigate specific pathways, other critical/sensitive periods for these exposures, as well as the mediating role of mental health risks at subsequent life stages. Efforts to reduce inequalities in adolescents’ mental health problems should focus on reducing socioeconomic inequalities and action to address the early years mediators identified in our study, particular on perinatal factors and family factors such as maternal mental health problems.

**What is already known on this subject**

- Adolescent mental health is in crisis in the UK, with stark inequalities and concerning signs of deterioration at a population level. Risk factors measured in the early years predict later mental health outcomes, but it is unclear how early years risk factors mediate social inequalities in adolescent mental health.

**What this study adds**

- Using robust methods for causal inference in observational data we show that around two-thirds of the social inequality in adolescent mental health is explained by early years risk factors identified by the age of 3 years.

**Policy implications**

- Public health policies to improve mental health in the UK should address modifiable socioeconomic inequalities and focus more on early years prevention.
