# Supplementary material for: How do early-life factors explain social inequalities in adolescent mental health? Findings from the UK Millennium Cohort Study
Source: J Epidemiol Community Health. 2019 Sep 6;73(11):1049–60. doi: 10.1136/jech-2019-212367 (PMC6877708; doi:10.1136/jech-2019-212367)

Online only supplementary material

How do early life factors explain social inequalities in adolescent mental health: Findings from the UK Millennium Cohort Study

Viviane S Straatmann, Eric TC Lai, Theis Lange, Melisa Campbell, Sophie Wickham, Anne-Marie Nybo-Andersen, Katrine Strandberg-Larsen, David Taylor-Robinson.

This data supplement contains additional information on the methods employed in the study. In addition, further plots and results are presented.

S1. Education System in the UK

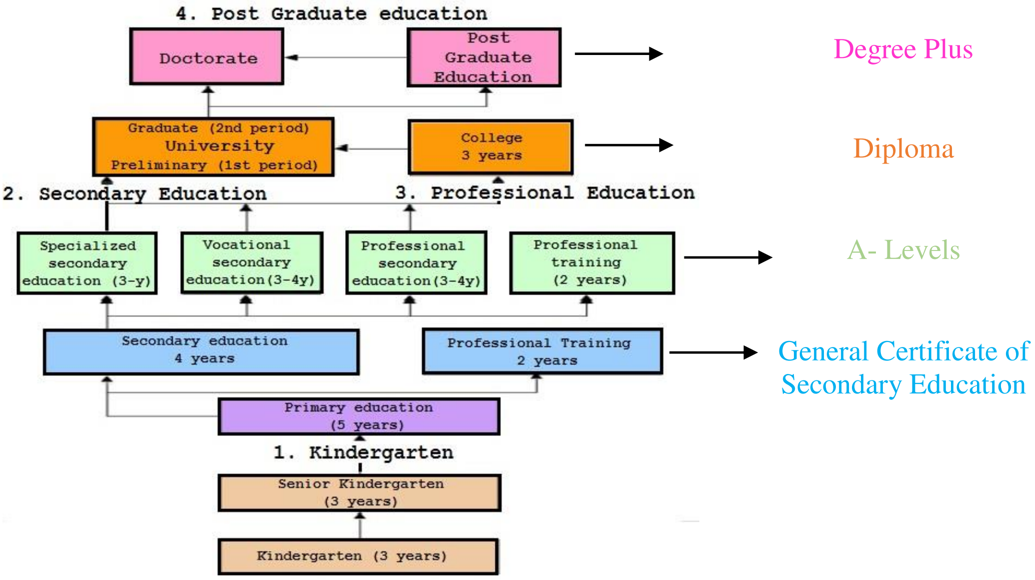

*By Razool [CC BY-SA 4.0 (https://creativecommons.org/licenses/by-sa/4.0)], from Wikimedia Commons

For more information access: *HM Government, 2011. Opening Doors, Breaking Barriers: a Strategy for Social Mobility. HM Government, London.*

S2. Coding of mediating variables

The full details of the coding of the mediators are provided below:

1. Perinatal factors (maternal smoking and consumption of alcohol in pregnancy, preterm gestational age, duration of breastfeeding lower than 4 months and low birth weight [5 items];
2. Child individual factors: delayed school readiness, long-term disabilities or illness, cognitive disabilities [3 items];
3. Family factors: maternal mental health problems, high levels of parent-child conflict, negligence or excessive rules of parenting style and lone parenthood [4 items];
4. Peer relation factors: time spent with friends per week, experience of being bullied or bullying other peers [3 items];
5. Neighbourhood factors: poor neighbourhood conditions and perceived safety [2 items].

1. Perinatal factors: we assessed perinatal data from the first sweep of the MCS, and categorized maternal smoking in pregnancy (‘*Gave up during pregnancy or did not smoke immediately prior to pregnancy versus smoked one or more a day*’), alcohol consumption in pregnancy (*Have you drank any alcohol during pregnancy*? ‘yes’ *versus* no’), preterm <37 weeks, duration of breastfeeding of at least 4 months, and low birth weight <2·5 kg into binary variables.

2. Child’s individual factors: data on child individual factors of health and development were assessed at age 3. School readiness was assessed using the Bracken School Readiness

Assessment (BSRA) ('average, advanced or very advanced' *versus* 'very delayed or delayed'). Cognitive disability was evaluated at age 3 through the British Ability Scale Second Edition (BAS II) Naming and Vocabulary, a validated standardised assessment of naming reasoning and knowledge, normed for children and adolescents from three years to 11years of age (Elliot, 1997). We defined children as having language disability if they scored –1·25 standard deviations below the normed mean score for the sample. The main responder was asked if the child had any disabilities or long-term illness ('no' *versus* 'yes').

Bracken B. Bracken Basic Concept Scale–Revised. San Antonio, TX: The Psychological Corporation 1998.

Elliott C. The British Ability Scales II. Windsor, Berkshire, 1996, UK: NFER-NELSON Publishing Company.

3. Family factors: family characteristics and parenting style were assessed when the child was age 3 years. We used the Kessler 6 (K6) scale to assess maternal mental health assessed at age 3. This measure asks in the last month how often respondents felt depressed, hopeless, restless or fidgety, worthless, or that everything was an effort. Respondents answered on a five-point scale from 1 (all the time) to 5 (none of the time). We reversed and rescaled all items from 0 to 4 for analysis purposes, so that high scores indicate high levels of psychological distress. We used validated cut offs, contrasting scores ('normal 0–5' *versus* 'high risk of distress 6–24') (Kessler et al., 2002). Parenting style assessed asking the main responder about her/his style of parenting at child age 3 and categorized it as 'firm discipline plus fun' *versus* 'education negligence or excess of rules'). We assessed child-parent relationship conflict at age 3 through the seven items using the Pianta Scale (Pianta, 1995) ('lowest conflict, score 7 to 15' *versus* 'highest conflict, score 16 to 40'). Lone parenthood was assessed at 9 months and coded as 'yes' *versus* 'no'.

Kessler RC, Andrews G, Colpe L, et al. Short screening scales to monitor population prevalence and trends in non-specific psychological distress. Psychol Med 2002; 32: 959–76.

Pianta RC. Child‐ Parent Relationship Scale. Charlottesville: University of Virginia, 1995.

4. Peer relations factors: Peer relations were measured at age 3 years. The main responder was asked about the amount of time that the child spends with friends ('spend any time peer week with friends'/ 'not at all'), and whether he/she has been bullied ('not being bullied' *versus* 'some true or certainly true') and or fights or bullies other peers ('not fights or bullies' *versus* 'some true or certainly true').

5. Neighbourhood factors: Information about neighbourhood conditions was asked for the main responder at the first sweep of the MCS and coded into 'not very common or not at all common neighbourhood problems' *versus* 'fairly or very common neighbourhood problems'. Responder was also asked about neighbourhood safety ('very safe or fairly safe' *versus* 'not safe') in the living area at age 3 years of child.

S3. Formulas and types of confounding

Natural direct effect (NDE): Ya,M(a*)– Ya*,M(a*)

This effect is the contrast between the counterfactual outcome if the individual were exposed at A = a, and the counterfactual outcome if the same individual were exposed at A = a*, with

the mediator assuming whatever value it would have taken at the reference value of the exposure A = a*.

Natural Indirect Effect (NIE): g (E (Y (aM (a))-Y (aM (a*))))

This effect is the contrast, having set the exposure at level A = a, between the counterfactual outcome if the mediator assumed whatever value it would have taken at a value of the exposure A = a and the counterfactual outcome if the mediator assumed whatever value it would have taken at a reference value of the exposure A = a*.

Total Effect (TE): g (E (Y (a)-Y (a*)))
The sum of the NDE and the NIE, even in presence of exposure-mediator interaction.

The four types of confounding are: (1) confounding of the exposure-outcome relationship; (2) confounding of the mediator-outcome relationship; (3) confounding of the exposure-mediator association; and (4) mediator-outcome confounders also affected by the exposure. For controlled direct effect, assumptions (1) and (2) are required. For the identification of natural direct and indirect effects, assumptions (3) and (4) are also needed.

S4. Full description of results presented in Table 2:

Adolescents of mothers with no qualifications were more than four times as likely to have had socioemotional behavioural problems than adolescents of mothers with degree level qualifications or higher (RR 4·48 [95%CI 2·91,6·88]). Younger maternal age at MCS birth, smoking in pregnancy, absence of alcohol consumption in pregnancy, less than 4 months of breastfeeding; cognitive disabilities, school readiness delayed, long term disabilities or illness at age 3; maternal mental health problem diagnosis, high conflicting relationship of children-parents, lone parenthood; being bullied or fights/bullies other peers; poorer neighbourhoods conditions and lower safety were statistically associated with an increased RR for adolescent mental health problems at age 14 years.

<u>S5.</u> Mediation Analysis With a Conterfactual Approach by Block of Risk Factors (Perinatal, Child Individual, Family, Peer Relations And Neighbourhood) in the Association of SECs (Relative Index Of Inequality (RII) of Maternal Education) and Adolescents Mental Health at Age 14 (Log-Relative Risk and Confidence Intervals [CI95%]) (N=6,509)

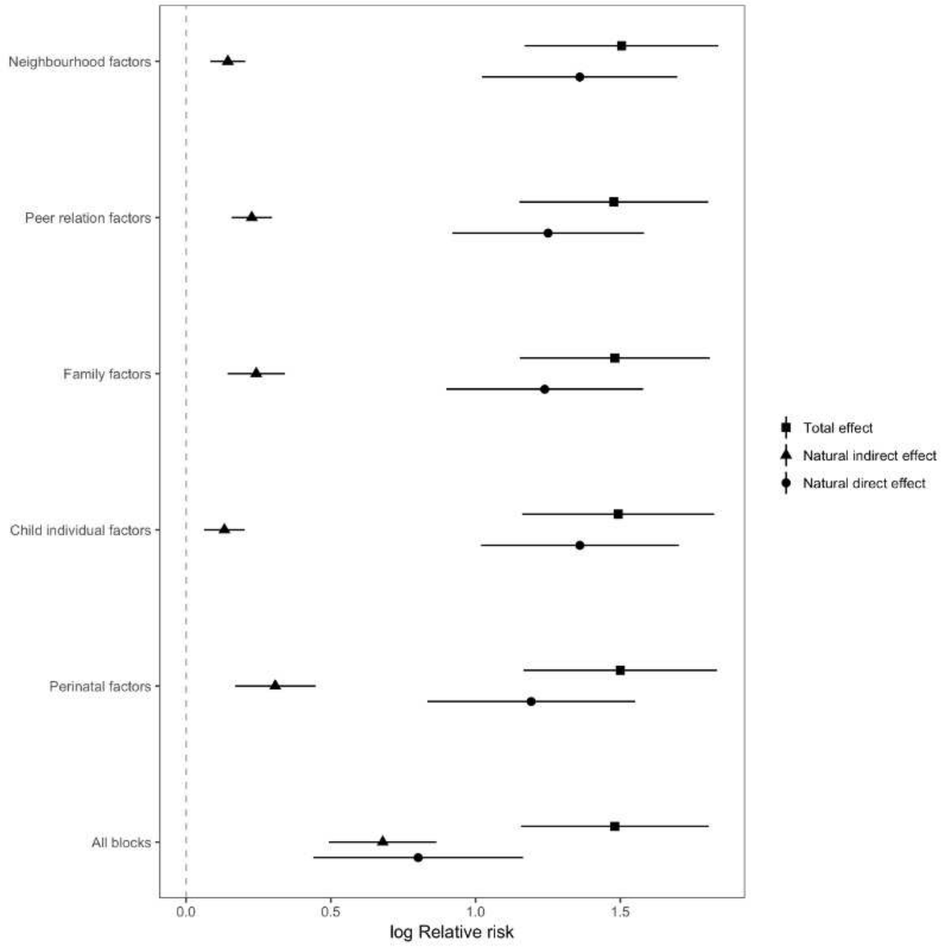

S6. Characteristics of the complete case study population, by level of maternal education at birth of child (N =6,509)- Full table

	Degree Plus	Diploma	A level	GCSE A-C	GCSE D-G	None	Total	p-value
	%	%	%	%	%	%	%	
**Adolescents socioemotional behavioural problems at age 14**	3·1	7·0	5·9	9·7	14·3	14·4	8·9	<0·001
**Child's sex**								0·701
Male	50·2	51·2	46·2	50·2	48·3	47·9	49·6	
Female	49·8	48·8	53·8	49·8	51·6	52·1	50·4	
**Maternal age at MCS birth**								<0·001
14-24	26·5	41·5	47·5	52·6	71·4	61·3	47	
25+	73·5	58·5	52·5	47·4	28·6	38·7	53	
**Maternal ethnicity**								<0·001
White	91·7	94·4	93	94·6	92·6	86·2	92·8	
Mixed	0·8	0·6	0	0·5	0·5	0·6	0·5	
Indian	2·0	1·4	1·2	0·9	1·4	2·0	1·4	
Pakistani	0·9	0·9	1·6	1·5	1·4	4·3	1·6	
Bangladeshi	0·1	0·4	0·3	0·2	0·4	1·0	0·3	
Black	2·4	2·3	0·5	0·6	1·5	0·8	1·2	
Other	1·4	0·7	1·0	0·3	0·1	1·0	0·7	
**Smoking in pregnancy**								<0·001
No	96·6	90·1	88·9	79·1	66·3	53·7	82·3	
Yes	3·4	9·9	11·1	20·9	33·7	46·3	17·7	
**Alcohol consume in pregnancy**								<0·001
None	34·7	48·4	54	59·7	69·1	73·7	53·9	
Any unit per week	65·3	51·6	46	40·3	30·1	26·3	46·1	
**Gestational age at birth**								0·007
Preterm	4·6	5·8	3·4	5·9	5·8	6·7	5·4	
Regular term	95·4	94·2	96·6	94·1	94·2	93·3	94·6	
**Child's birth weight**								<0·001
Low weight	3·6	5·4	4·7	5·5	6·4	8·9	5·3	
Normal+	96·4	94·6	95·3	94·5	93·6	91·1	94·7	
**Breastfeeding at least 4 months**								<0·001
Yes	64·3	40·3	41·9	26·9	17·9	15·8	37·2	
No	35·7	59·7	58·1	73·1	82·1	84·2	62·8	
**Cognitive disability at age 3**								<0·001
No	98·5	97·7	97·7	96	94·2	88·9	96·2	
Yes	1·5	2·3	2·3	4	5·7	11·1	3·8	
**School readiness at age 3**								<0·001
Average, advanced or very advanced	96·6	96·1	95·1	91·4	85·4	79·7	92·1	
Very delayed or delayed	3·4	3·9	4·9	8·6	14·6	20·3	7·9	
**Child long term disabilities or illness at age 3**								0·063
No	85·7	85·2	88·7	84·6	83·7	80·6	85	
Yes	14·3	14·8	11·3	15·4	16·3	19·4	15	
**Maternal mental health problems**								<0·001

No	89·1	87·3	86·5	81·6	81·6	70·6	83·7	
Yes	10·9	12·7	13·5	18·4	18·4	29·4	16·3	
**Parenting style**								<0·001
Firm discipline plus fun	59·9	56·3	54·6	47·1	42	37·4	51	
Negligent or harsh parenting style	40·1	43·7	45·4	52·1	58	62·6	49	
**Child-parents conflict relationship**								0·001
Low conflicts	47·5	47·5	47·8	47·7	45·5	36·5	46·5	
High conflicts	52·5	52·5	52·2	52·3	54·5	63·5	53·5	
**Lone Parenthood**								<0·001
No	98·3	95·6	92	87·5	83·7	76·2	90·2	
Yes	1·7	4·4	8	12·5	16·3	23·8	9·8	
**Child's time spend with friends**								0·027
Any time per week with friends	99·4	100	98·5	99·4	99·4	98·7	99·3	
Not at all	0·6	0	1·5	0·6	0·6	1·3	0·7	
**Being bullied**								<0·001
Not being bullied	95·7	96·1	94·3	93·5	89·5	86·6	93·5	
Some true or certainly true	4·3	3·9	5·7	6·5	10·5	13·4	6·5	
**Fights or bullies other peers**								<0·001
Not fights or bullies	90·7	89·3	88·4	85·6	78·4	69·6	84·1	
Some true or certainly true	9·3	10·7	11·6	14·4	21·6	30·4	15·9	
**Neighbourhood conditions**								<0·001
Not at all or nor very common neighbourhood problems	50	44·1	43·7	39·8	29·2	27·4	41·1	
Fairly or very common neighbourhood problems	50	55·9	56·3	60·2	70·8	72·6	58·9	
**Neighbourhood safety**								<0·001
Very safe	95·1	93·3	90·5	85·5	81·9	76·4	87	
Fairly safe	5·1	6·7	9·5	14·5	18·1	23·6	13	

S7-S14. Robustness tests

We used multiple imputation by chained equation in order to check whether there are differences in descriptive and associative results of complete cases and imputed samples (S7-S9). However, given the complexity of the methods applied for mediation analysis, we did not multiply impute the data for the mediation analysis, and for comparison all analyses were presented for the complete case sample in the main document.

S7. Prevalence (%) of Adolescents Mental Health Problems in the UK at Age 14 by Maternal Education at Birth (N=9,962)

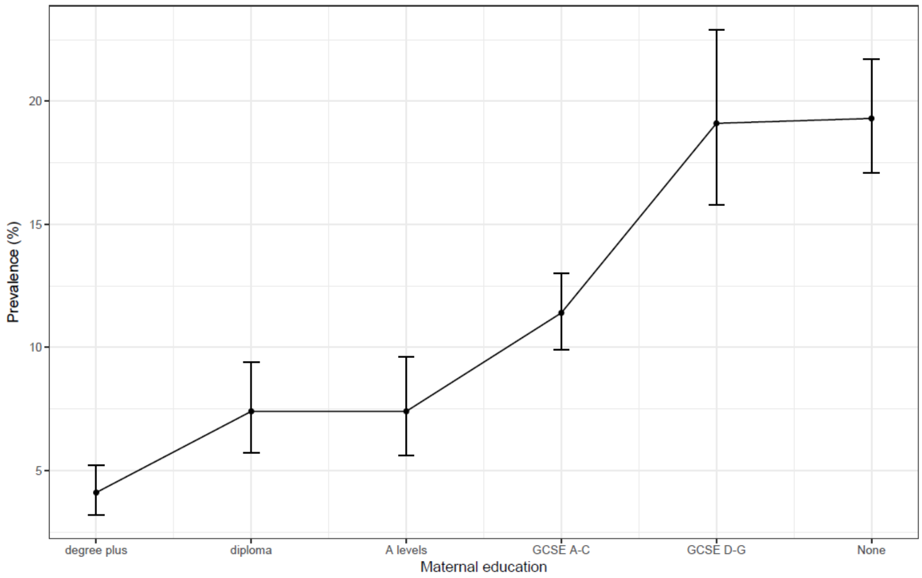

S8. Univariate Relative Risk (RR) (95% CI) of Mental Health Problems at Age 14 and Prevalence by Maternal Education at Birth

Maternal education	RR (95% CI)	Adolescents with mental health problems %
		11·4 (10·5,11·2)
Degree plus	1·00	3·5
Diploma	2·10 (1·50,2·95)	7·6
A levels	1·94 (1·33,2·84)	7
GCSE A-C	2·89 (2·20,3·80)	10·4
GCSE D-G	4·88 (3·56,6·69)	17·6
None	5·09 (3·89,6·64)	18·3
**Child's sex**		
Male	1·33 (1·15,1·55)	-
Female	1·00	-
**Maternal age at MCS birth**		
14-24	1·74 (1·46,2·08)	-
25+	1·00	-
**Maternal ethnicity**		
White	1·00	-
Mixed	1·20 (0·70, 1·16)	-
Indian	0·62 (0·40, 0·69)	-
Pakistani	1·21 (0·95, 1·38)	-
Bangladeshi	0·87 (0·78, 1·08)	-
Black	0·98 (0·75, 1·26)	-
Other	0·69 (0·37, 1·66)	-
**Smoking in pregnancy**		
No	1·00	-
Yes	1·92 (1·61,2·29)	-

**Alcohol consume in pregnancy**		
None	1·00	-
Any unit per week	0·68 (0·56,0·81)	-
**Gestational age at birth**		
Preterm	1·58 (1·20,2·08)	-
Regular term	1·00	-
**Child's birth weight**		
Low weight	1·52 (1·15,2·00)	-
Normal+	1·00	-
**Breastfeeding at least 4 months**		
Yes	1·00	-
No	2·17 (1·81,2·60)	-
**Cognitive disability at age 3**		
No	1·00	-
Yes	1·82 (1·40,2·37)	-
**School readiness at age 3**		
Average, advanced or very advanced	1·00	-
Very delayed or delayed	1·96 (1·59,2·43)	-
**Child long term disabilities or illness at age 3**		
No	1·00	-
Yes	1·56 (1·29,1·90)	-
**Maternal mental health problems**		
No	1·00	-
Yes	2·20 (1·85,2·62)	-
**Parenting style**		
Firm discipline plus fun	1·00	-
Education negligence or excess of rules	1·24 (1·04,1·49)	-
**Child-parents conflict relationship**		
Low conflicts	1·00	-
High conflicts	2·35 (1·88,2·95)	-
**Lone Parenthood**		
No	1·00	-
Yes	1·86 (1·53,2·25)	-
**Child's time spend with friends**		
Any time per week with friends	1·00	-
Not at all	2·00 (1·28,3·12)	-
**Being bullied**		
Not being bullied	1·00	-
Some true or certainly true	1·99 (1·54,2·57)	-
**Fights or bullies other peers**		
Not fights or bullies	1·00	-
Some true or certainly true	2·71 (2·29,3·20)	-
**Neighbourhood conditions**		
Not at all or nor very common neighbourhood problems	1·00	-
Fairly or very common neighbourhood problems	1·56 (1·27,1·93)	-
**Neighbourhood safety**		
Very safe	1·00	-
Fairly safe	1·76 (1·45,2·15)	-

S9. Regression Models for Mental Health Problems at Age 14 by blocks of Risk Factors (N =9,962)

		RR (95%CI)*						
		**Baseline****	**Model 1**	**Model 2**	**Model 3**	**Model 4**	**Model 5**	**Model 6**
**Maternal education**	**Diploma**	1·98 (1·40,2·79)	1·77 (1·25,2·50)	1·96 (1·39,2·75)	1·98 (1·37,2·85)	1·97 (1·40,2·78)	1·94 (1·37,2·73)	1·77 (1·23,2·55)
	**A levels**	1·81 (1·23,2·65)	1·61 (1·09,2·37)	1·79 (1·22,2·62)	1·63 (1·08,2·45)	1·79 (1·23,2·63)	1·78 (1·21,2·61)	1·53 (1·01,2·31)
	**GCSE A-C**	2·61 (1·97,3·45)	2·10 (1·56,2·82)	2·47 (1·86,3·27)	2·36 (1·73,3·21)	2·49 (1·88,3·28)	2·46 (1·85,3·27)	1·88 (1·35,2·60)
	**GCSE D-G**	4·18 (3·02,5·79)	3·16 (2·25,4·44)	3·79 (2·72,5·28)	3·34 (2·38,4·69)	3·65 (2·64,5·05)	3·83 (2·76,5·13)	2·30 (1·58,3·34)
	**None**	4·57 (3·44,6·06)	3·27 (2·42,4·42)	3·90 (2·90,5·23)	3·55 (2·56,4·92)	3·62 (2·71,4·84)	4·17 (3·12,5·57)	2·20 (1·54,3·14)
**Perinatal Factors**								
Smoking in pregnancy			1·30 (1·07,1·58)	-	-	-	-	1·22 (0·99,1·49)
Alcohol consume in pregnancy			0·87 (0·73,1·04)	-	-	-	-	0·93 (0·77,1·10)
Gestational age at birth			1·35 (1·00,1·84)	-	-	-	-	1·24 (0·91,1·67)
Child's birth weight			1·12 (0·81,1·53)	-	-	-	-	1·04 (0·74,1·46)
Breastfeeding at least 4 months			1·35 (1·01,1·80)	-	-	-	-	1·20 (0·96,1·50)
**Child Individual Factors**								
Cognitive disability			-	1·32 (0·97,2·75)	-	-	-	1·40 (1·06,1·84)
School readiness			-	1·38 (1·09,1·74)	-	-	-	1·18 (0·95,1·45)
Child long term disabilities or illness			-	1·49 (1·23,1·80)	-	-	-	1·41 (1·16,1·69)
**Family Factors**								
Maternal mental health problems			-	-	1·61 (1·34,1·93)	-	-	1·43 (1·19,1·72)
Parenting style			-	-	1·09 (0·92,1·29)	-	-	1·06 (0·90,1·25)
Lone Parenthood			-	-	1·21 (0·98,1·48)	-	-	1·08 (0·89,1·33)
Child-parents conflict relationship			-	-	1·94 (1·59,2·38)	-	-	1·66 (1·36, 2·04)
**Peer Relations Factors**								
Child's time spend with friends			-	-	-	1·82 (1·16,2·84)	-	1·60 (0·94,2·71)
Being bullied			-	-	-	1·46 (1·13,1·88)	-	1·24 (0·95,1·60)
Fights or bullies other peers			-	-	-	2·11 (1·78,2·51)	-	1·71 (1·44,2·04)
**Neighbourhood Factors**								
Neighbourhood conditions			-	-	-	-	1·25 (1·02,1·54)	1·21 (0·99,1·48)
Neighbourhood safety			-	-	-	-	1·39 (1·13,1·70)	1·19 (0·95,1·48)
**Proportion attenuated (%) *****			36·4	18·7	28·5	26·6	11·2	57·4

*All models were adjusted for baseline confounders (maternal age birth, child sex and maternal ethnicity) - omitted table results;
**Adjusted only for baseline confounders - omitted table results;
*** Proportion of RR attenuated by comparison of Baseline Model with Models 1-6

Baseline cases and complete cases sociodemographic characteristic are show in S10.

S10. Baseline (Child's Sex N= 10,246; Maternal Ethnicity N= 9,497; Maternal Education N=10,296) and Complete Case (N=6,509) Characteristics.

	Baseline (% and CI 95%)	Complete cases (% and CI 95%)
**Child's sex**		
Female	49.6 (48.5,50.6)	48.8 (47.6, 50.0)
Male	50.4 (49.4, 51.5)	51.2 (50.0, 52.4)
**Maternal ethnicity**		
White	87.8 (86.4, 87.7)	93.3 (92.6, 93.8)
Mixed	1.0 (0.7, 1.5)	0.5 (0.4, 1.2)
Indian	1.9 (1.4, 2.7)	1.1 (0.8, 1.4)
Pakistani	3.4 (2.2, 5.4)	1.9 (1.7, 3.1)
Bangladeshi	1.1 (0.6, 1.9)	0.6 (0.4, 1.2)
Black	3.3 (2.3, 4.6)	1.7 (1.3, 2.6)
Other	1.5 (1.1, 2.2)	0.9 (0.8, 1.7)
**Maternal education**		
Degree plus	21.2 (20.4, 22.1)	23.8 (22.7, 24.8)
Diploma	10.1 (9.5, 10.7)	11.2 (10.5, 12.0)
A levels	10.5 (9.9, 11.2)	11.5 (10.8, 12.3)
GCSE A-C	33.6 (32.6, 34.5)	35.2 (34.1, 36.4)
GCSE D-G	9.8 (9.2, 10.5)	8.9 (8.3, 9.7)
None	14.7 (14.0, 15.5)	9.4 (8.7, 10.1)

We repeated our first step regression analysis using RII as the exposure variable for the purposes of comparison with the counterfactual mediation analysis. The pattern of results was similar using RII as the exposure for the first step of the analysis (S11).

S11. Regression Models, Using Relative Index of Inequality (RII), for Mental Health Problems at Age 14 (N =6,509)

		RR (95%CI)*						
		**Baseline****	**Model 1**	**Model 2**	**Model 3**	**Model 4**	**Model 5**	**Model 6**
**Maternal education**	**RII**	4·16 (2·80,6·17)	2·73 (1·76,4·23)	3·63 (2·42,5·44)	3·29 (2·14,5·08)	3·12 (2·12,4·60)	3·64 (2·40,5·49)	1·89 (1·17,3·03)
**Perinatal Factors**								
Smoking in pregnancy			1·46 (1·11,1·92)	-	-	-	-	1·21 (0·92,1·59)
Alcohol consume in pregnancy			0·89 (0·73,1·09)	-	-	-	-	0·90 (0·73,1·10)
Gestational age at birth			0·93 (0·64,1·36)	-	-	-	-	0·87 (0·58,1·30)
Child's birth weight			1·16 (0·77,1·75)	-	-	-	-	1·16 (0·77,1·75)
Breastfeeding at least 4 months			1·35 (1·01,1·79)	-	-	-	-	1·26 (0·94,1·68)
**Child Individual Factors**								
Cognitive disability			-	1·62 (1·10,2·37)	-	-	-	1·46 (1·00,2·12)
School readiness			-	1·20 (0·86,1·60)	-	-	-	1·01 (0·72,1·39)
Child long term disabilities or illness			-	1·59 (1·24,2·03)	-	-	-	1·47 (1·15,1·88)
**Family Factors**								
Maternal mental health problems			-	-	1·62 (1·28,2·06)	-	-	1·54 (1·20,1·95)
Parenting style			-	-	1·02 (0·82,1·26)	-	-	1·06 (0·82,1·24)
Lone Parenthood			-	-	1·27 (0·94,1·70)	-	-	1·14 (0·84,1·53)
Child-parents conflict relationship			-	-	2·15 (1·66,2·77)	-	-	1·84 (1·42,2·37)
**Peer Relations Factors**								
Child's time spend with friends			-	-	-	1·88 (0·93,3·79)	-	1·68 (0·88,3·21)
Being bullied			-	-	-	1·37 (0·96,1·96)	-	1·15 (0·79,1·66)
Fights or bullies other peers			-	-	-	2·23 (1·76,2·82)	-	1·73 (1·36,2·21)
**Neighbourhood Factors**								
Neighbourhood conditions			-	-	-	-	1·51 (1·19,1·93)	1·38 (1·08,1·74)
Neighbourhood safety			-	-	-	-	1·16 (0·85,1·58)	1·04 (0·77,1·40)
**Proportion attenuated (%) *****			45·3	16·8	27·5	32·9	16·5	71·8

*All models were adjusted for baseline confounders (maternal age birth, child sex and maternal ethnicity) - omitted table results;
**Adjusted only for baseline confounders - omitted table results;
*** Proportion of RR attenuated by comparison of Baseline Model with Models 1-6

<u>S12.</u> Regression models for socioemotional behavioural problems at age 14 using probabilistic chain approach*. Covariate estimates using complete case analysis (N =6,509)

					RR (95%CI)*			
		**Baseline**	**Model 1**	**Model 2**	**Model 3**	**Model 4**	**Model 5**	**Model 6**
**Maternal education**	**Diploma**	2·08 (1·32,3·27)	1·88 (1·19,2·98)	1·90 (1·21,3·00)	1·89 (1·19,2·97)	1·89 (1·19,3·00)	1·87 (1·18,2·99)	1·89 (1·20,3·00)
	**A levels**	1·71 (1·03,2·85)	1·55 (0·92,2·59)	1·51 (0·99,2·60)	1·58 (0·96,2·65)	1·57 (0·96,2·65)	1·02 (0·03,2·60)	1·55 (0·93,2·60)
	**GCSE A-C**	2·70 (1·83,3·98)	2·21 (1·46,3·33)	2·19 (1·43,3·27)	2·07 (1·40,3·15)	2·06 (1·42,3·20)	2·15 (1·44,3·16)	2·02 (1·32,3·11)
	**GCSE D-G**	3·73 (2·41,5·77)	2·79 (1·74,4·49)	2·76 (1·72,4·40)	2·50 (1·44,4·01)	2·48 (1·46,3·99)	2·70 (1·66,4·31)	2·36 (1·44,3·87)
	**None**	3·82 (2·48,5·88)	2·67 (1·68,4·23)	2·60 (1·60,4·22)	2·40 (1·37,4·27)	2·36 (1·30,4·20)	2·50 (1·51,4·16)	1·99 (1·22,3·26)
**Perinatal Factors**								
Smoking in pregnancy			1·47 (1·12,1·92)	1·42 (1·08,1·76)	1·28 (0·96,1·72)	1·26 (0·95,1·72)	1·43 (1·10,1·73)	1·23 (0·94,1·61)
Alcohol consume in pregnancy			0·92 (0·74,1·13)	0·92 (0·73,1·10)	0·91 (0·74,1·10)	0·91 (0·74,1·12)	0·91 (0·74,1·11)	0·90 (0·74,1·10)
Gestational age at birth			0·95 (0·65,1·38)	0·94 (0·62,1·31)	0·89 (0·60,1·31)	0·89 (0·64,1·32)	0·94 (0·62,1·31)	0·87 (0·58,1·29)
Child's birth weight			1·16 (0·77,1·76)	1·18 (0·79,1·80)	1·17 (0·77,1·76)	1·17 (0·77,1·76)	1·17 (0·78,1·77)	1·17 (0·78,1·75)
Breastfeeding at least 4 months			1·35 (1·01,1·80)	1·31 (0·99,1·75)	1·27 (0·96,1·68)	1·29 (1·98,1·68)	1·33 (1·01,1·78)	1·21 (0·91,1·61)
**Child Individual Factors**								
Cognitive disability			-	1·48 (1·05,2·23)	-	-	-	1·45 (0·99,2·12)
School readiness			-	1·06 (0·79,1·58)	-	-	-	1·01 (0·73,1·40)
Child long term disabilities or illness			-	1·53 (1·18,1·98)	-	-	-	1·47 (1·15,1·87)
**Family Factors**								
Maternal mental health problems			-	-	1·56 (1·27,3·02)	-	-	1·52 (1·19,1·93)
Parenting style			-	-	1·00 (0·78,1·18)	-	-	1·02 (0·83,1·25)
Lone Parenthood			-	-	1·23 (0·90,1·66)	-	-	1·15 (0·85,1·55)
Child-parents conflict relationship			-	-	2·13 (1·52,2·73)	-	-	1·85 (1·43,2·39)
**Peer Relations Factors**								
Child's time spend with friends			-	-	-	1·84 (0·92,3·78)	-	1·70 (0·91,3·20)
Being bullied			-	-	-	1·28 (0·89,1·88)	-	1·15 (0·79,1·67)
Fights or bullies other peers			-	-	-	2·05 (1·60,2·70)	-	1·76 (1·39,2·23)
**Neighbourhood Factors**								
Neighbourhood conditions			-	-	-	-	1·47 (1·10,1·88)	1·35 (1·07,1·72)
Neighbourhood safety			-	-	-	-	1·16 (0·81,1·59)	1·05 (0·78,1·41)
**Proportion attenuated (%) *** **			40·8	43·3	50·3	51·7	46·8	64·8

*All models were adjusted for baseline confounders (maternal age birth, child sex and maternal ethnicity) - omitted table results; **Adjusted only for baseline confounders - omitted table results; *** Proportion of RR attenuated by comparison of Baseline Model with Models 1-6.

Model 1: adjusted for perinatal factors; Model 2: adjusted for perinatal and child individual factors; Model 3: adjusted for perinatal and family factors; Model 4: adjusted for perinatal and peer relation factors; Model 5: adjusted for perinatal and neighbourhood factors; Model 6: mutually adjusted for all blocks of risk factors.

<u>S13.</u> Slope index of inequality (SII) (scaling maternal education from 0 to 1, with a higher score representing higher SECs) using self-reported depressed mood (Mood and Feelings Questionnaire-MFQ) at age 14.

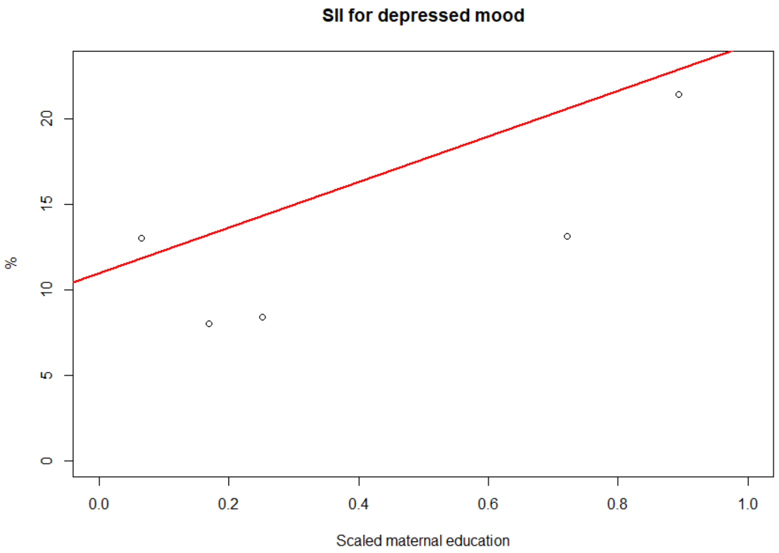

S14. Baseline and adjusted regression models using depressed mood at age 14 as the outcome.

		RR (95%CI)*	
		**Baseline***	**Model 6**
**Maternal education**	**Diploma**	1·15 (0·92,1·42)	1·08 (0·85,1·37)
	**A levels**	1·14 (0·92,1·40)	1·10 (0·89,1·38)
	**GCSE A-C**	1·20 (1·01,1·42)	1·09 (0·92,1·30)
	**GCSE D-G**	1·22 (0·96,1·55)	1·03 (0·79,1·31)
	**None**	1·24 (1·02,1·52)	1·08 (0·82,1·27)
**Perinatal Factors**			
Smoking in pregnancy			1·36 (1·17,1·59)
Alcohol consume in pregnancy			0·95 (0·88,1·17)
Gestational age at birth			0·84 (0·66,1·13)
Child's birth weight			1·17 (0·78,1·75)
Breastfeeding at least 4 months			1·07 (0·94,1·22)
**Child Individual Factors**			
Cognitive disability			1·33 (0·99,2·20)
School readiness			1·03 (1·01,1·56)
Child long term disabilities or illness			1·32 (1·06,1·89)
**Family Factors**			
Maternal mental health problems			1·35 (1·17,1·56)
Parenting style			1·07 (0·97,1·17)
Lone Parenthood			1·31 (1·15,1·50)
Child-parents conflict relationship			1·89 (1·33,2·11)
**Peer Relations Factors**			
Child's time spend with friends			1·58 (0·92,3·13)
Being bullied			1·07 (0·94,1·20)
Fights or bullies other peers			1·18 (0.98·,1·16)
**Neighbourhood Factors**			
Neighbourhood conditions			1·12 (1·03,1·25)
Neighbourhood safety			1·02 (0·56,1·14)
**Proportion attenuated (%) ****			66·7

*Adjusted only for baseline confounders - omitted table results;

** Proportion of RR attenuated by comparison of Baseline Model with Model 6 (adjusted by all blocks of risk factors)

S15. Bias analysis for causal mediation analysis

**Natural direct effect (RR = 2.18)**			**Natural indirect effect (RR = 1.94)**		
Prevalence of binary unmeasured confounder (%)			Prevalence of binary unmeasured confounder (%)		
High maternal education (RII = 1)	Low maternal education (RII = 0)	OR required to explain away the observed effect*	High maternal education (RII = 1)	Low maternal education (RII = 0)	OR required to explain away the observed effect*
5	5	.	5	5	.
5	10	.	5	10	.
5	20	33.02	5	20	.
5	40	7.45	5	40	0.18
5	60	5.94	5	60	.
5	80	1.7	5	80	2.20
10	5	.	10	5	6.68
10	10	.	10	10	.
10	20	.	10	20	.
10	40	15.13	10	40	0.30
10	60	5.65	10	60	0.49
10	80	3.19	10	80	0.33
20	5	.	20	5	2.17
20	10	.	20	10	3.23
20	20	.	20	20	.
20	40	.	20	40	0.54
20	60	.	20	60	.
20	80	6.99	20	80	0.76
40	5	.	40	5	1.87
40	10	.	40	10	1.13
40	20	.	40	20	2.25
40	40	.	40	40	.
40	60	0.30	40	60	.
40	80	.	40	80	1.13
60	5	.	60	5	0.67
60	10	.	60	10	1.18
60	20	.	60	20	1.83
60	40	.	60	40	6.10
60	60	.	60	60	.
60	80	.	60	80	0.13
80	5	.	80	5	.
80	10	0.10	80	10	1.08
80	20	.	80	20	0.44
80	40	.	80	40	1.41
80	60	.	80	60	37.89

*Odds ratios ≤ 0 were not presented.

The second step (counterfactual mediation analysis) was also repeated using equivalised family income as an alternative measure of childhood SECs (S16)- see below:

<u>S16.</u> Mediation Analysis With a Conterfactual Approach by Block of Risk Factors (Perinatal, Child Individual, Family, Peer Relations and Neighbourhood) in the Association of RII of Income and Adolescents Mental Health at Age 14 (Relative Risk and Confidence Intervals [CI95%])

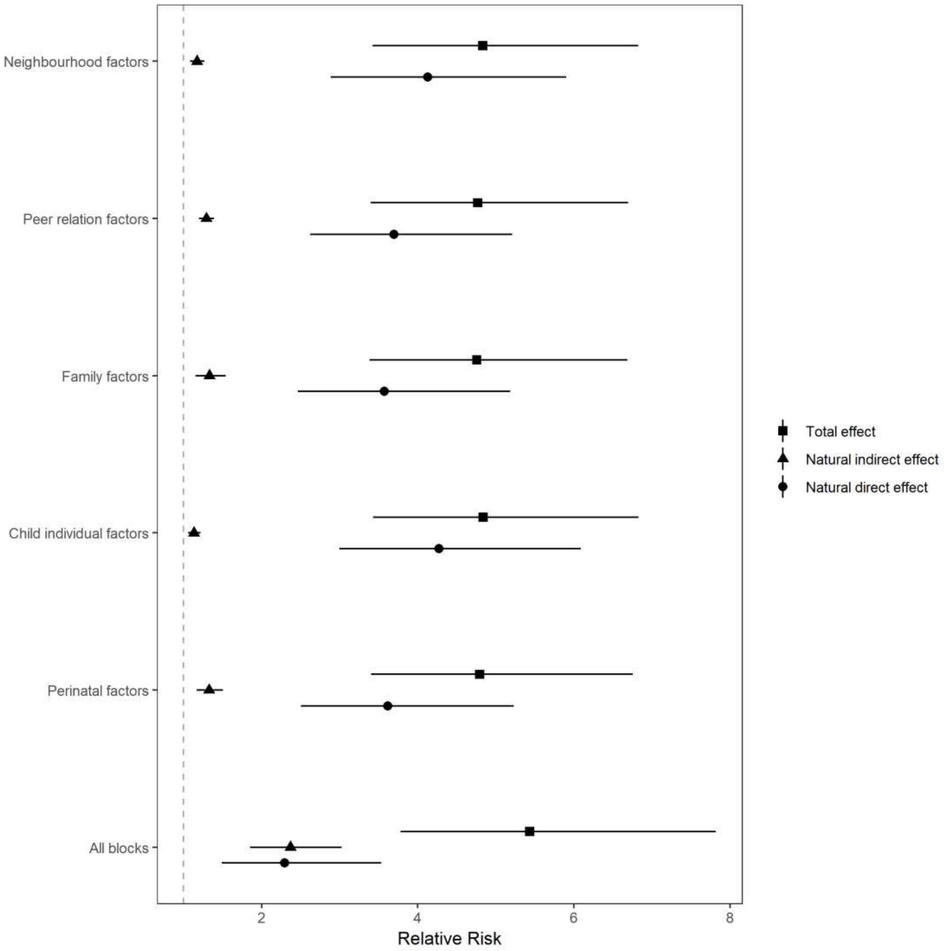

Supplement: Supplementary data [file jech-2019-212367supp001.pdf]
